# Genome-wide assessment and mapping of inbreeding depression identifies candidate genes associated with semen traits in Holstein bulls

**DOI:** 10.1186/s12864-023-09298-1

**Published:** 2023-05-03

**Authors:** Mohammad Ghoreishifar, Seyed Milad Vahedi, Siavash Salek Ardestani, Majid Khansefid, Jennie E. Pryce

**Affiliations:** 1 Agriculture Victoria Research, AgriBio, Centre for AgriBioscience, 5 Ring Road, Bundoora, Victoria 3083 Australia; 2 School of Applied Systems Biology, La Trobe University, Bundoora, Victoria 3083 Australia; 3 Department of Animal Science and Aquaculture, Dalhousie University, Truro, NS B2N5E3 Canada; 4 Department of Animal Science, University of Zanjan, Zanjan, 4537138791 Iran

**Keywords:** Inbreeding depression, Runs of homozygosity (ROH), Male fertility, Sperm quality, Dairy cattle

## Abstract

**Background:**

The reduction in phenotypic performance of a population due to mating between close relatives is called inbreeding depression. The genetic background of inbreeding depression for semen traits is poorly understood. Thus, the objectives were to estimate the effect of inbreeding and to identify genomic regions underlying inbreeding depression of semen traits including ejaculate volume (EV), sperm concentration (SC), and sperm motility (SM). The dataset comprised ~ 330 K semen records from ~ 1.5 K Holstein bulls genotyped with 50 K single nucleotide polymorphism (SNP) BeadChip. Genomic inbreeding coefficients were estimated using runs of homozygosity (i.e., *F*_ROH_ > 1 Mb) and excess of SNP homozygosity (*F*_SNP_). The effect of inbreeding was estimated by regressing phenotypes of semen traits on inbreeding coefficients. Associated variants with inbreeding depression were also detected by regressing phenotypes on ROH state of the variants.

**Results:**

Significant inbreeding depression was observed for SC and SM (*p* < 0.01). A 1% increase in *F*_ROH_ reduced SM and SC by 0.28% and 0.42% of the population mean, respectively. By splitting *F*_ROH_ into different lengths, we found significant reduction in SC and SM due to longer ROH, which is indicative of more recent inbreeding. A genome-wide association study revealed two signals positioned on BTA 8 associated with inbreeding depression of SC (*p* < 0.00001; FDR < 0.02). Three candidate genes of *GALNTL6*, *HMGB2*, and *ADAM29*, located in these regions, have established and conserved connections with reproduction and/or male fertility. Moreover, six genomic regions on BTA 3, 9, 21 and 28 were associated with SM (*p* < 0.0001; FDR < 0.08). These genomic regions contained genes including *PRMT6*, *SCAPER*, *EDC3*, and *LIN28B* with established connections to spermatogenesis or fertility.

**Conclusions:**

Inbreeding depression adversely affects SC and SM, with evidence that longer ROH, or more recent inbreeding, being especially detrimental. There are genomic regions associated with semen traits that seems to be especially sensitive to homozygosity, and evidence to support some from other studies. Breeding companies may wish to consider avoiding homozygosity in these regions for potential artificial insemination sires.

**Supplementary Information:**

The online version contains supplementary material available at 10.1186/s12864-023-09298-1.

## Background

A reduction in the mean performance of a quantitative trait owing to mating between relatives is known as inbreeding depression. The genetic background of inbreeding depression is increased homozygosity because of descending two copy of the same allele from a common ancestor [1]. Inbreeding depression has traditionally been investigated by regressing phenotypes on pedigree-derived inbreeding coefficients [2]. Nowadays the accessibility of single nucleotide polymorphism (SNP) genotypes, distributed throughout the genome, enables researchers to estimate inbreeding coefficient (*F*) based on genomic data and provides them with some benefits. Genomic inbreeding does not depend on the quality and completeness of the pedigree [3], and is expected to be more accurate than pedigree-based inbreeding because of considering Mendelian sampling effect [4]. Genomic inbreeding coefficients can be estimated by SNP-based methods (e.g., excess of homozygosity), or alternatively, by runs of homozygosity (ROH) [5]. Genomic ROH-based inbreeding has the merit of providing an estimation regarding the age of inbreeding by distinguishing between identical by state (IBS) and identical by descent (IBD) segments [6, 7].

The approximate age of inbreeding can be estimated by the $E\left({L}_{IBD-H}|gcA\right)=\frac{100}{2gcA}$ equation, where $E\left({L}_{IBD-H}|gcA\right)$ is the expected length of an IBD haplotype given $gcA$, which is the number of generations from a common ancestor [8]. Indeed, shorter ROHs display more ancient inbreeding, while longer ROHs are indicative of inbreeding events that have occurred in recent generations [6, 9]. Recent inbreeding is considered to have a more significant adverse effect compared with ancient inbreeding. This phenomenon is explained by genetic purging, in that selection operates against deleterious alleles, because most of them are recessive or partially recessive [10, 11]. Consequently, through selection, the frequency of favourable alleles increases in the population. Only a few experiments have studied the impact of recent and old inbreeding on livestock performance, and the findings were sometimes conflicting [12, 13].

Another relative merit of ROH-based inbreeding is that it enables researchers to identify genes and genomic regions underlying inbreeding depression. However, only a few researchers have used ROH to do so. Pryce et al. [14] used a statistical model to identify genes and genomic regions contributing to inbreeding depression of calving interval and milk yield in dairy cattle. Ferenčaković et al. [15] applied a similar model to find the associated regions with semen quality traits in beef cattle. These authors concluded that inbreeding depression could be reduced by avoiding the mating which would result in homozygous offspring for deleterious haplotypes causing inbreeding depression [14, 15].

Recent studies have shown that inbreeding influences many quantitative traits in livestock including fertility (e.g., Doekes et al. [16]). Fertility is a complex trait being influenced by both female and male components as well as the interaction between them [17]. However, male component of fertility has largely been ignored, probably because of the assumption that the artificial insemination (AI) centres monitor and standardize the quality of semen before it is distributed [18, 19]. However, this comes at a cost to the AI company and in extreme circumstances may mean that bulls are not marketable at all. In the dairy cattle industry, one bull is usually mated with thousands of cows. Therefore, using bulls with improved male fertility can immediately affect the overall herd fertility [18]. Non-return rates (NRR) are generally used as indicators of male fertility as data are abundantly available, whereas sperm quality data is usually the domain of AI companies. A moderate to high genetic and phenotypic correlation between semen quality traits and service sire NRR has been reported [20]. For many AI companies semen quality traits are used as a screening tool (i.e., this data is available on most sires), while only those bulls that pass quality checks end up with NRR data.

Based on this background information, the objectives of the current study were: (i) to estimate genomic inbreeding coefficients and inbreeding depression; (ii) to explore inbreeding depression resulted from old and recent inbreeding; and (iii) to identify genetic variants contributing to inbreeding depression, for semen quality traits in Holstein bulls.

## Material and methods

### Data description

Phenotypic records of three semen quality traits including EV, SC, and SM belonging to 1,508 Holstein bulls genotyped using the Illumina Bovine SNP50 BeadChip were used in this study. This dataset was published in a study by Yin et al. [21]. Phenotypic records were already collected from bulls born between 1996 and 2016. Briefly, the semen quality traits were obtained from 12 AI centres across China [21]. EV (in ml) was read directly from a graduated collection tube. SC was measured using a spectrophotometer as 10^8^ spermatozoa per ml [22]. SM was estimated as the proportion of forward-moving sperm by microscopic examination by experienced technicians [21]. Phenotypic records were filtered to eliminate possible outliers from the analysis. After quality control, a total of 332,531 records ranged between 1 and 25 mL for EV, 330,270 records ranged between 1 and 30 × 10^8^ mL for SC, and 330,199 records for SM ranged between 0.1 and 0.98, remained for further analyses. Across the three semen quality traits, the minimum and maximum number of records per bull were 6 and 1,415, respectively.

A total of 52,886 SNPs across 29 autosomes were available in the initial dataset. There were no missing genotypes in the dataset as they were already imputed [21]. Genotype data was converted to ARS-UCD1.2 reference genome [23] using liftOver tool (https://genome.ucsc.edu/cgi-bin/hgLiftOver) and the default options. In this process, 231 SNPs were removed, which were duplicated or with unknown positions in ARS-UCD1.2 reference genome; therefore, 52,655 SNPs remained for the following analyses.

### Genomic inbreeding coefficients

All SNPs, even those with very low minor allele frequency (MAF) were used to calculate inbreeding as suggested by Meyermans et al. [24]. Two estimates of genomic *F were used*.

#### Inbreeding coefficient based on excess of homozygosity (FSNP)

*F*_SNP_ was defined as the excess of homozygosity [7], which is estimated as follows:

$${{\mathrm{F}}_{\mathrm{SNP}}}_{\mathrm{i}}=\frac{({\mathrm{OH}}_{\mathrm{i}}-\mathrm{EH})}{(\mathrm{s}-\mathrm{EH})}$$

in which, $\mathrm{s}$ is the total number of SNPs, ${\mathrm{OH}}_{\mathrm{i}}$ (observed homozygosity) in the ${\mathrm{animal}}_{\mathrm{i}}$ equals to ${\sum }_{\mathrm{j}=1}^{\mathrm{s}}{\mathrm{X}}_{\mathrm{ij}}$, in which ${\mathrm{X}}_{\mathrm{ij}}$ is 0 or 1 if the ${\mathrm{animal}}_{\mathrm{i}}$ for ${\mathrm{SNP}}_{\mathrm{j}}$ is heterozygous or homozygous, respectively. The expected homozygosity in the population (i.e., $\mathrm{EH}$) can be calculated as ${\sum }_{\mathrm{j}=1}^{\mathrm{s}}\left[1-2{\mathrm{m}}_{\mathrm{j}}(1-{\mathrm{m}}_{\mathrm{j}})\right]$, where ${\mathrm{m}}_{\mathrm{j}}$ shows the MAF for ${\mathrm{SNP}}_{\mathrm{j}}$. *F*_SNP_ can take positive or negative values when $\mathrm{EH}$ is smaller or larger than ${\mathrm{OH}}_{i}$, respectively.

#### Inbreeding coefficients based on runs of homozygosity (FROH)

This inbreeding coefficient represents the proportion of the genome that is covered with ROH segments, and is calculated as follows [5]:

$${F}_{{\mathrm{ROH}}_{\mathrm{i}}}=\frac{{\sum }_{\mathrm{k}=1}^{\mathrm{n}}{\mathrm{L}}_{{\mathrm{ROH}}_{\mathrm{ik}}}}{{\mathrm{L}}_{\mathrm{g}}}$$

where $\mathrm{n}$ shows the total number of ROH identified for the ${\mathrm{animal}}_{\mathrm{i}}$, ${\mathrm{L}}_{{\mathrm{ROH}}_{\mathrm{ik}}}$ represents the length of the ${\mathrm{ROH}}_{\mathrm{k}}$ identified for the ${\mathrm{animal}}_{\mathrm{i}}$, and ${\mathrm{L}}_{\mathrm{g}}$ is the length of the autosomal genome covered by SNPs. The scanning approach implemented in the PLINK 1.9 [25] software was used. In this study we used similar parameters to those used by Doekes et al. [12]. To define ROH we allowed: (a) a minimum ROH length of 1 Mb; (b) a minimum number of 15 SNP per ROH; (c) an average SNP density of 1 SNP per 100 Kb; (d) a maximum of one heterozygous call within a ROH; and (e) a maximum gap of 500 kb between adjacent SNPs.

To study the contribution of length of ROH on inbreeding depression, we classified ROH into five groups: (i) 1 to 2 Mb, (ii) 2 to 4 Mb, (iii) 4 to 8 Mb, (iv) 8 to 16 Mb, and (v) > 16 Mb (abbreviated to *F*_ROH1*−2*_,* F*_ROH2*−4*_, *F*_ROH4*−*8_, *F*_ROH8−16_, and *F*_ROH>16_ respectively). Inbreeding coefficients relevant to different length classes were calculated by summing up ROH segments of that class for each animal divided by ${\mathrm{L}}_{\mathrm{g}}$.

### Data quality control and model fitting

The SNPs with MAF < 0.01 and Hardy–Weinberg equilibrium chi-square test *p* value < 1e^−6^ were discarded using PLINK 1.9 [25]. We utilised the same thresholds employed in the study by Yin et al. [21], as we analysed publicly available data provided by the authors. A total of 43,886 SNPs passed the quality control steps. These SNPs were used to construct the genomic relationship matrix (**G**) to fit the inbreeding depression models. We adapted the model of Yin et al. [21] and included inbreeding coefficients (*F*_ROH_ or *F*_SNP_) in the model to study the effect of inbreeding depression on semen traits:

1 $${\mathrm{y}}_{\mathrm{ijklm}}=\upmu +{\mathrm{year}\_\mathrm{season}}_{\mathrm{i}}+{\mathrm{insemin}\_\mathrm{center}}_{\mathrm{j}}+{\mathrm{interval}}_{\mathrm{k}}+{\mathrm{n}\_\mathrm{sample}}_{\mathrm{l}}+{\mathrm{b}}_{1}\times {\mathrm{age}}_{\mathrm{ijklm}}+{\mathrm{b}}_{2}\times {F}_{\mathrm{m}}+{\mathrm{animal}}_{\mathrm{m}}+{\mathrm{perm}}_{\mathrm{m}}+{\mathrm{e}}_{\mathrm{ijklm}}$$

where, ${\mathrm{y}}_{\mathrm{ijklm}}$ is the dependent variable (i.e., phenotypic records of EV, SC, and SM), $\upmu$ is the overall mean, ${\mathrm{year}\_\mathrm{season}}_{\mathrm{i}}$ is the combined fixed effect of the ${\mathrm{i}}^{\mathrm{th}}$ year and season (60 levels), ${\mathrm{insemin}\_\mathrm{center}}_{\mathrm{j}}$ is fixed effect of the ${\mathrm{j}}^{\mathrm{th}}$ AI centre (12 levels), ${\mathrm{interval}}_{\mathrm{k}}$ is fixed effect of the interval between two subsequent semen collections (four levels), ${\mathrm{n}\_\mathrm{sample}}_{\mathrm{l}}$ is fixed effect of the number of sample collections on the respective collection day (three levels), ${\mathrm{animal}}_{\mathrm{m}}$ and ${\mathrm{perm}}_{\mathrm{m}}$ are, respectively, the random effects and permanent environment of the ${\mathrm{m}}^{\mathrm{th}}$ bull; and ${\mathrm{e}}_{\mathrm{ijklm}}$ is the random error term. In this model, ${\mathrm{b}}_{1}$ is the regression coefficient on ${\mathrm{age}}_{\mathrm{ijklm}}$ (i.e., age of the ${\mathrm{m}}^{\mathrm{th}}$ bull in months at the time of phenotype recording); and ${\mathrm{b}}_{2}$ is the regression coefficient on ${F}_{\mathrm{m}}$ (i.e., inbreeding coefficient of the ${\mathrm{m}}^{\mathrm{th}}$ bull). The bull effect was assumed to follow N(0,$\mathbf{G}{\upsigma }_{\mathrm{u}}^{2}$), where $\mathbf{G}$ is the genomic relationship matrix, and ${\upsigma }_{\mathrm{u}}^{2}$ is additive genetic variance.

### Model fitting of different ROH length

*F*_ROH1*−2*_,* F*_ROH2*−4*_, *F*_ROH4*−*8_, *F*_ROH8−16_, and *F*_ROH>16_ were fitted simultaneously in the model, as follows:

2 $${\mathrm{y}}_{\mathrm{ijklm}}=\upmu +{\mathrm{year}\_\mathrm{season}}_{\mathrm{i}}+{\mathrm{insemin}\_\mathrm{center}}_{\mathrm{j}}+{\mathrm{interval}}_{\mathrm{k}}+{\mathrm{n}\_\mathrm{sample}}_{\mathrm{l}}+{\mathrm{b}}_{1}{\times \mathrm{age}}_{\mathrm{ijklm}}+\sum_{\mathrm{q}=1}^{\mathrm{n}=5}{(\mathrm{b}}_{2\mathrm{q}}\times {F}_{\mathrm{mq}})+{\mathrm{animal}}_{\mathrm{m}}+{\mathrm{perm}}_{\mathrm{m}}+{\mathrm{e}}_{\mathrm{ijklm}}$$

This model is similar to the Model (1) except $\sum_{\mathrm{q}=1}^{\mathrm{n}=5}{(\mathrm{b}}_{2\mathrm{q}}\times {\mathrm{F}}_{\mathrm{mq}})$, in which ${\mathrm{b}}_{2\mathrm{q}}$ represents the regression coefficient of the ${\mathrm{q}}^{\mathrm{th}}$ inbreeding class and ${\mathrm{F}}_{\mathrm{mq}}$ is the inbreeding coefficient of the ${\mathrm{m}}^{\mathrm{th}}$ bull for that ROH length class.

### Mapping genomic regions associated with inbreeding depression

SC and SM were used in the association analyses since we found significant inbreeding (i.e., *F*_ROH_) depression for these traits in Model (1). To map genomic regions underlying inbreeding depression, each of the autosomal SNPs (*n* = 52,655) were fitted one at a time and sequentially in the model (3):

3 $${\mathrm{y}}_{\mathrm{ijklm}}=\upmu +{\mathrm{year}\_\mathrm{season}}_{\mathrm{i}}+{\mathrm{insemin}\_\mathrm{center}}_{\mathrm{j}}+{\mathrm{interval}}_{\mathrm{k}}+{\mathrm{n}\_\mathrm{sample}}_{\mathrm{l}}+{\mathrm{b}}_{1}{\times \mathrm{age}}_{\mathrm{ijklm}}+{\mathrm{b}}_{2}{ \times \mathrm{SNP}}_{\mathrm{m}}+{\mathrm{b}}_{3}{\times \mathrm{ROH}}_{\mathrm{m}}+{\mathrm{animal}}_{\mathrm{m}}+{\mathrm{perm}}_{\mathrm{m}}+{\mathrm{e}}_{\mathrm{ijklm}}$$

which is similar to the model (1), but two new covariates were fitted instead of *F*: (i) the regression coefficient ${\mathrm{b}}_{2}$ on ${\mathrm{SNP}}_{\mathrm{m}}$ which represents the SNP genotype for the ${\mathrm{m}}^{\mathrm{th}}$ bull and was included to adjust for the additive genetic effect of the presence of one more copy of the same allele. Thus, ${\mathrm{SNP}}_{\mathrm{m}}$ was coded as 0 for homozygous genotypes (AA) or 2 for alternative homozygous genotypes (aa) and coded as 1 for heterozygous genotypes (Aa); (ii) the regression coefficient ${\mathrm{b}}_{3}$ on ${\mathrm{ROH}}_{\mathrm{m}}$, which represents ROH state of the SNP on bull m, was coded as 1 when the ${\mathrm{SNP}}_{\mathrm{m}}$ is present within an ROH and coded as 0 otherwise. Thus, after correcting for the additive effect of the SNP, the effect of homozygosity was estimated at that position.

We used AIREMLF90 1.148 [26] to estimate the variance components and fit the above models. To investigate whether *F*_SNP_ and *F*_ROH_, in the model (1), *F*_ROH1−2_, *F*_ROH2−4_, *F*_ROH4−8_, *F*_ROH8−16_, and *F*_ROH>16_ in the model (2), and ${\mathrm{ROH}}_{\mathrm{m}}$ in the model (3) had a statistically significant adverse effect on semen traits, we estimated the regression coefficients and corresponding standard errors (SE) for inbreeding measures. A t-test was then conducted in R [27] to test the significance of the regression coefficients.

Model (3) was run for each individual SNP at a time because the aim was to map genomic regions associated with inbreeding depression. To consider multiple testing problem, a method proposed by Bolormaa et al. [28] was used to find FDR for the *p* value significance threshold. Assuming $\mathrm{FDR}=\frac{\mathrm{P}(1-\mathrm{N})}{\mathrm{N}(1-\mathrm{P})}$, where P is the *p* value threshold, and N is the proportion of significant SNPs. Different *p* value thresholds were tested to find the *p* value with the minimum FDR for SC and SM.

## Results and discussion

### Descriptive statistics and heritability

The descriptive statistics and estimated heritability and repeatability are shown in Table 1. Heritability estimates for EV, SM, and SC were 0.13 ± 0.02, 0.08 ± 0.02, and 0.11 ± 0.02, respectively. The heritability was estimated using a GBLUP (i.e., model 1) while excluding the inbreeding effect from the model. To estimate variance components, we used the priors which were reported by using a BLUP model in the study by Yin et al. [29]. Our heritability estimates were comparable to the pooled mean heritability estimates (± SE) obtained from across different studies, which were 0.2 (0.02), 0.05 (0.01) and 0.17 (0.03), for semen volume, sperm motility and concentration respectively [30].

**Table 1:** Descriptive statistics and the estimated heritability (h^2^) and repeatability (r) of the three semen quality traits

Trait (unit)	N ^a^	Mean (SD)	Min	Max	h^2^ ± SE ^b^	r ± SE ^b^
Ejaculate volume	332,531	6.76 (2.97)	1	25	0.13 ± 0.02	0.28 ± 0.01
Sperm concentration	330,270	12.67 (4.61)	1	30	0.11 ± 0.02	0.32 ± 0.01
Sperm motility	330,199	0.67 (0.16)	0.1	0.98	0.08 ± 0.02	0.28 ± 0.01

^a^ Number of records

^b^ ${h}^{2}=\frac{{\upsigma }_{\mathrm{a}}^{2}}{{\upsigma }_{\mathrm{a}}^{2}+{\upsigma }_{\mathrm{pe}}^{2}+{\upsigma }_{\mathrm{e}}^{2}}$, and $r=\frac{{\upsigma }_{\mathrm{a}}^{2}+{\upsigma }_{\mathrm{pe}}^{2}}{{\upsigma }_{\mathrm{a}}^{2}+{\upsigma }_{\mathrm{pe}}^{2}+{\upsigma }_{\mathrm{e}}^{2}}$; where ${\upsigma }_{\mathrm{a}}^{2}$ is additive genetic variance, ${\upsigma }_{\mathrm{pe}}^{2}$ is permanent environment variance, and ${\upsigma }_{\mathrm{e}}^{2}$ is residual variance

### Inbreeding coefficients

**Fig. 1:**
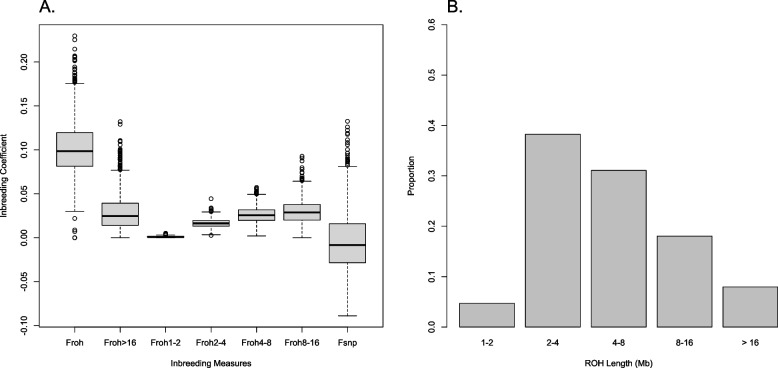
Box plot of the estimated inbreeding coefficients (**a**); and proportion of different ROH length (**b**) Footnote: Seven *F*_SNP_ values between -0.1 and -0.3 in Fig. 1a were not shown

**Fig. 2:**
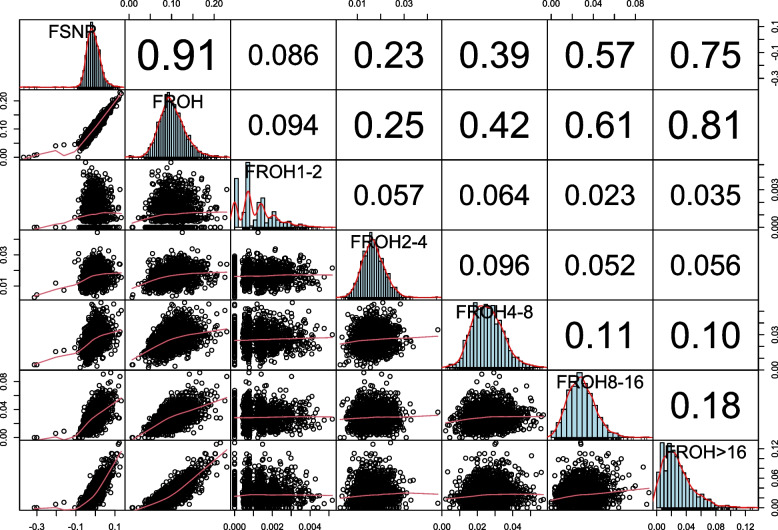
Correlogram of Pearson correlations between different inbreeding measures. The genomic inbreeding coefficients are estimated based on *F*_SNP_; *F*_ROH_; *F*_ROH 1-2 Mb_; *F*_ROH 2-4 Mb_; *F*_ROH 4-8 Mb_; *F*_ROH 8-16 Mb_; and *F*_ROH > 16 Mb_

Figure 1a shows the average ± SD of *F*_SNP_, *F*_ROH_, *F*_ROH1−2_, *F*_ROH2−4_, *F*_ROH4−8_, *F*_ROH8−16_, and *F*_ROH>16_, and Fig. 1b represents the distribution of the proportion of different lengths of ROH class. The mean inbreeding coefficient of *F*_ROH_ was 0.09 ± 0.02. In a previous study by Bjelland et al. [31] *F*_ROH_ of 0.04 ± 0.02 was reported for US Holstein cows, which was less than the amount we obtained for Holstein bulls of China. The average ± SD of *F*_SNP_ -0.006 ± 0.03 in our study was less than *F*_ROH_ (Fig. 1a). This is because *F*_SNP_ can also be negative when the observed homozygosity is lower than the expected homozygosity. However, we observed a strong correlation of 0.91 between *F*_SNP_ and *F*_ROH_ (Fig. 2). Our finding was in line with previous works; in which, a correlation of 0.96 and 0.86 were reported between *F*_ROH_ and *F*_SNP_ in pigs [32] and sheep [33], respectively. The high correlation between *F*_ROH_ and *F*_SNP_ can probably be caused by the fact that a large amount of the total inbreeding captured by *F*_SNP_ was related to SNPs in ROH state. Keller et al. [7] suggested that as Ne decreases, the similarity between molecular inbreeding measures (e.g., *F*_ROH_ and *F*_SNP_) increases. Therefore, the difference in the correlation reported by previous studies in pig and sheep breeds [32, 33] compared with our correlation (i.e., 0.96 and 0.86 versus 0.91) is more likely to be due to differences in Ne.

Recent inbreeding (i.e., *F*_ROH>16 Mb_) had the highest correlation with *F*_SNP_ (0.75) and *F*_ROH_ (0.81), while more distant inbreeding (i.e., *F*_ROH1-2 Mb_) had the lowest correlation (~ 0.09) with the same measures (Fig. 2). The high correlation between recent and total inbreeding was in accordance with previous reports in Holstein cattle [12, 13] and pig [32]. According to Fig. 1b, ROH of short length (i.e., 2–4 Mb) had the highest frequency. However, it seems that only a small proportion of *F*_ROH_ is explained by *F*_ROH1-2 Mb_ and *F*_ROH2-4 Mb_ (Fig. 1a). Therefore, longer ROHs raised from more recent inbreeding covered a higher proportion of the genome compared with the short ROHs, and contributed more to the total inbreeding. This could explain the higher correlation between total and recent inbreeding to some extent.

### Inbreeding depression of semen quality traits

In this study, we hypothesized that inbreeding would negatively affect semen quality traits. Inbreeding depression estimates of the semen traits are shown in Table 2. Neither *F*_SNP_ nor *F*_ROH_ was significant for EV (*p* > 0.05). However, both *F*_ROH_ and *F*_SNP_ had significant effects on SM (*p* < 0.01). A 1% increase in *F*_ROH_, reduces SM by 0.28% of the mean population. Inbreeding depression for SM has been documented in livestock species including cattle [2, 34], sheep [33], as well as in horses [35, 36], endangered ungulates [37] and zebra finch [38]. Dorado et al. [34] studying the impact of inbreeding on sperm quality found features in sperm motility (i.e., erratic tracks, unexpected change in direction, non-progressive but highly active spermatozoa) in highly inbreed bulls that were associated with hyperactivate spermatozoa pattern [39, 40]. In inbred mice, Carey and Olds-Clarke [41] reported an increase in epididymal motility of spermatozoa (i.e., early sperm hyperactivation) which was accompanied by a reduction in fertility. A similar case was also reported in donkeys where males having a higher proportion of hyperactive spermatozoa were less fertile [42]. Therefore, it is plausible that transportation of premature hyperactive spermatozoa along the lower female reproductive tract and the spermatozoas’ access to the fertilization spot could be impaired in inbred animals, leading to a reduction in fertility.

**Table 2:** Regression coefficients and standard errors (SE) of different inbreeding measures on the three semen traits

Inbreeding measure	Ejaculate volume	Sperm concentration	Sperm motility
	mL	1e-8 per mL	%
*F*_ROH_	-1.63 (1.25)	-5.33 (2.3)**	-0.19 (0.05)**
*F*_ROH 1–2 Mb_	63.2 (88.9)	-95.04 (134.1)	-1.78 (4.20)
*F*_ROH 2–4 Mb_	9.04 (17.5)	-17.58 (26.3)	1.16 (0.80)
*F*_ROH 4–8 Mb_	15.6 (9.46) *	-15.14 (14.2)	0.67 (0.40)
*F*_ROH 8–16 Mb_	3.95 (6.2)	-15.57 (9.3)*	0.17 (0.30)
*F*_ROH >16 Mb_	-7.15 (3.9) *	-3.45 (5.9)	-0.53 (0.20)**
*F*_SNP_	-0.17 (0.96)	-1.68 (1.8)	-0.15 (0.04) **

^*^Shows significance level at *p* < 0.05; and ** at *p* < 0.01

Our result show that the effect of *F*_ROH_ on SC was significantly different from zero (*p* < 0.01), and a 1% increase in *F*_ROH_ decreased SC about 0.42% of the population mean (Table 2). In a dairy sheep breed, Antonios et al. [33] observed that the effect of *F*_ROH_ on SC was null. However, in Oldfield mice (*Peromyscus polionotus*), significant effect of inbreeding depression on testicular SC was reported [43]. To the extent of our knowledge, the relationship between shift in sperm count and fertility has not been studied in livestock species. In human, this relationship was largely depended on the median SC and onset and type of shift such that a dramatic decline from the high level slightly changed the fertility, whereas a minor decay from the low level remarkably deteriorated fertility [44]. A similar conclusion was also drawn in a captive population of mice [43]. Therefore, considering the adverse effect of inbreeding on sperm count or concentration, it seems to become a challenge in populations with endangered species or populations with small Ne, which are subject to some strong levels of inbreeding.

### ROH length and inbreeding depression

We also hypothesized that recent inbreeding would have a more detrimental effect on SM and SC. All the ROH length classes were simultaneously fitted in the model to account for the correlations between classes [33]. No significant inbreeding depression was found for the old ROH classes of length 1–2 Mb and 2–4 Mb. These ROH classes represent a common ancestor back to about 50–25 and 25–12.5 generations ago, respectively. However, we found a significant adverse effect of recent inbreeding (i.e., long ROH) on the SM and SC. The effect of *F*_ROH>16 Mb_ was significant on SM (*p* < 0.01) and the effect of *F*_ROH8-16 Mb_ was significant on SC (*p* < 0.05). This level of inbreeding traces back to common ancestors approximately 3 and 6 to 3 generations ago, respectively. Our findings regarding the importance of more recent inbreeding on semen traits were in line with previous studies on semen and other traits. Antonios et al. [33] reported significant effects of recent inbreeding (i.e., ROH > 17 Mb) on SM, while old inbreeding (i.e., ROH 4–17 Mb) was not significant. Makanjuola et al. [13] studied inbreeding depression on production and fertility traits in Holstein dairy cows. They reported that recent inbreeding had a greater detrimental effect, while old inbreeding had a favourable effect on the studied traits. Our results could be explained by the purging effect of deleterious alleles in older generations. Genetic purging occurs when long-lasting selection for a given trait causes the harmful alleles to be eliminated [45]. Studying inbreeding depression in a Holstein–Friesian population, Mc Parland et al. [46] observed genetic purging effects for the production traits (i.e., milk, fat, and protein); but not for female fertility traits (i.e., age at first calving, calving interval, and survival). Doekes et al. [12] showed a purging effect of inbreeding depression for most of the milk production and female fertility traits by using pedigree-based inbreeding coefficients. Unfortunately, we were unable to confirm the purging effect since the pedigree data only went back one generation. In contrast to our findings regarding the importance of recent inbreeding, Doekes et al. [12] reported that both old and recent components of inbreeding can cause inbreeding depression across production and female fertility traits. The inconsistency between Doekes et al. [12] and our results may be due to differences in the genetic architecture of the traits studied. Moreover, there may be different representations of high-use sires, which are typically used in artificial insemination to produce large numbers of offspring, and therefore can have a disproportionate impact on the genetic makeup of a population.

### Identification of genomic regions contributing to inbreeding depression

**Fig. 3:**
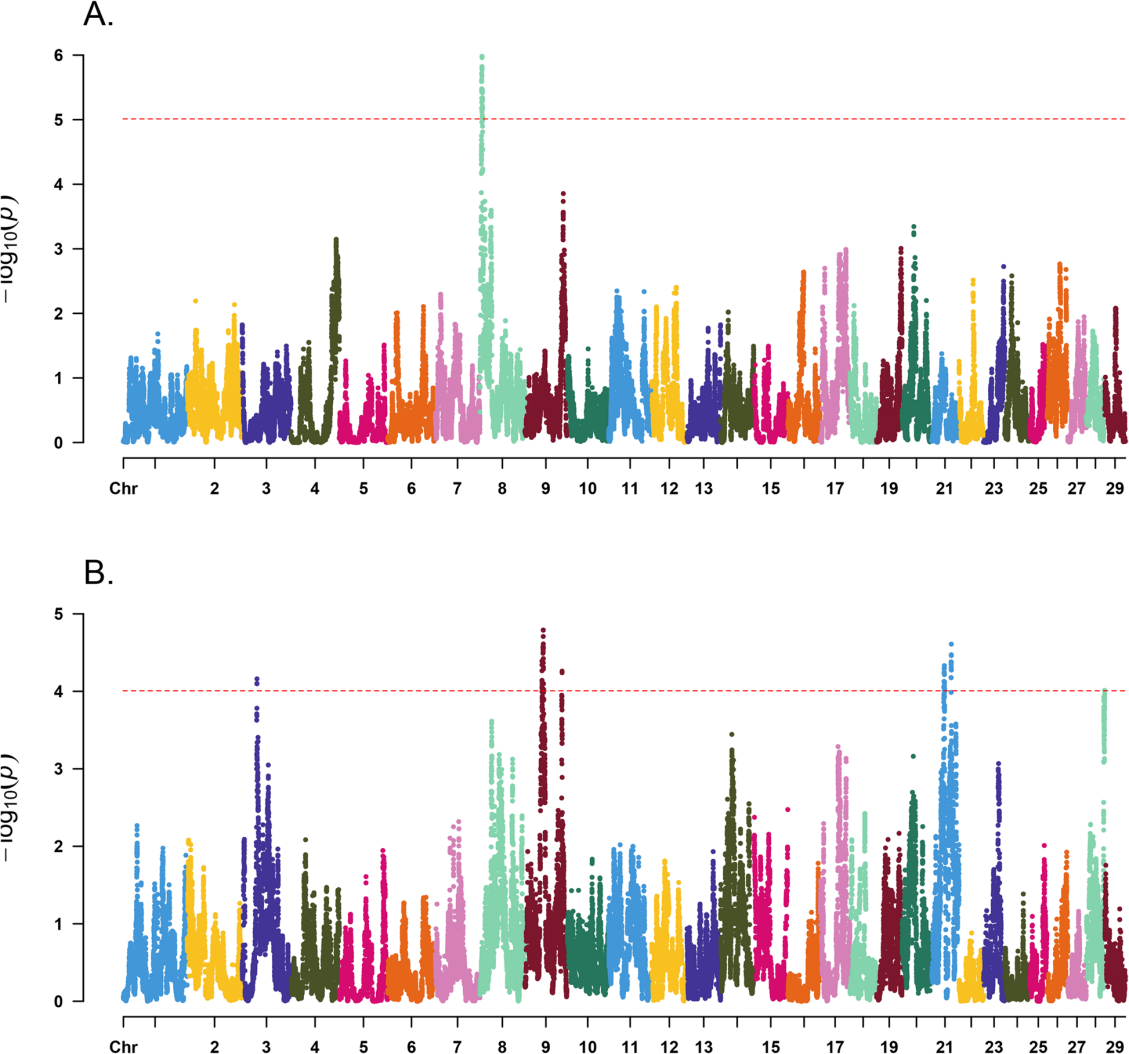
Manhattan plots of -log10 *p* values, illustrating inbreeding depression of sperm concentration (*p* < 0.00001; FDR < 0.02) (**a**); and sperm motility (*p* < 0.0001; FDR < 0.08) (**b**)

To link inbreeding depression of semen traits to genomic regions, a statistical test was performed for every autosomal SNP, considering the potential additive effect and ROH state of the SNP in the model. The distributions of -log_10_ *p* values for the association between SNP in ROH state and inbreeding depression for semen quality traits across chromosomes are shown in Fig. 3.

Identification of genomic regions contributing to inbreeding depression of female fertility and semen quality traits has already been reported in cattle [14, 15]. Yet, to our knowledge, this is the first study to apply the same approach to sperm quality traits in dairy cattle. We calculated the FDR for each trait at various *p* value thresholds. As shown in Fig. 3a, there were two signals positioned at 4.21–4.66 Mb and at 5.02–6.74 Mb on BTA 8. These inbreeding depression signals contained 52 SNP significantly associated (*p* < 0.00001; FDR < 2%) with the SC trait (Additional file 1 Table S1). There were 12 protein coding genes within 500 Kb upstream and downstream of these regions: *GALNTL6*, *GALNT7*, *HMGB2*, *SAP30*, *SCRG1*, *HAND2*, *FBXO8*, *CEP44*, *HPGD*, *GLRA3*, and *ADAM29*. Three candidate genes of *GALNTL6*, *HMGB2*, and *ADAM29* have established connections with reproduction and/or male fertility. *GALNTL6* is a member of membrane-bound polypeptide N-acetyl galactosaminyl transferase family that catalyses the first step in mucin-type O-glycosylation of peptides in the Golgi apparatus. The *GALNTL6* gene was reported as a candidate gene for sperm characteristics in Holstein dairy cattle [47]. Parker Gaddis et al. [48] did a genome-wide association and gene network analyses of Holstein female fertility traits, the largest gene network in their study included 24 genes, with a regulatory role of *GALNTL6* gene. The same authors mentioned that among those 24 genes, *GALNTL6* was significantly associated with cow and heifer conception rate and daughter pregnancy rate. *HMGB2* (High Mobility Group Box 2) is a member of the HMGB protein family, which includes the ubiquitous HMGB1 and the embryo-specific HMGB3. It has been reported in mice that adult males lacking *HMGB2* (Hmgb2^−^/^−^) have reduced fertility, that correlates with Sertoli and germ cell degeneration in seminiferous tubules and immotile spermatozoa [49]. The protein encoded by *ADAM29* is highly expressed in the testis and involved in human spermatogenesis [50]. Gene Ontology related to this gene include spermatogenesis GO:0,007,283 and male gonad development GO:0,008,584. In addition, three genes of *ADAM29*, *GLRA3* and *HPGD* were reported to be associated with early pregnancy in Nelore heifers [51]. The functions of the remaining genes are currently unknown, and further investigation is needed to determine their role in male fertility.

We also identified inbreeding depression signals with significant adverse effects on SM trait (Fig. 3b; Additional file 2 Table S2). These regions were in clusters on BTA 3 (36.7–36.9), BTA 9 (42.3–43.2 Mb, 44.8–46.3 Mb, and 92.1–92.2 Mb), BTA 21 (31.6–31.7 Mb), and an obvious peak on BTA 28, with the most significant SNP (i.e., 28:45,649,746) passing our significance threshold criterion. Overall, 71 SNPs were significantly associated with ROH-based inbreeding depression for SM (*p* < 0.0001; FDR < 8%). There were 74 genes within the 500 Kb upstream and downstream of the significant SNPs such as *PRMT6*, *SCAPER*, *EDC3* and *LIN28B* (Additional file 3 Table S3). *PRMT6* (Protein Arginine Methyltransferase 6) on BTA 3 at the region 36.73–36.94 is highly expressed in testis and influences cell migration and apoptosis of germ cells (http://www.genecards.org/) [52]. Gene Ontology annotations related to *PRMT6* include methyltransferase activity and protein methyltransferase activity, and the diseases associated with *PRMT6* include male infertility in humans [52–54]. *PRMT6* was previously reported to be associated with sperm concentration in Holstein bulls (*P* = 6.5E-6) [55]. In the present study, however, it was significantly associated with inbreeding depression of SM. We found a significant gene on BTA 21 at the region 31.6–31.7 Mb. The *SCAPER* (S Phase Cyclin A-Associated Protein in The Endoplasmic Reticulum) gene in this region is highly expressed in testis and involved in spermatogenesis (GO: 0,007,283) (http://www.genecards.org/). *SCAPER*, originally identified as a cell cycle regulator, was also suggested to be a ciliary protein. It has been reported to be associated with sterility in male and reduced fertility in female mice [56]. The same authors hypothesized that *SCAPER* is a crucial component in both the male and female reproductive systems [56]. In human patients homozygous for a mutation which disturbs *SCAPER* expression in spermatogonia are azoospermic due to early defects in spermatogenesis [56].

There were also other genes associated with inbreeding depression of SC in our study which could be associated with male fertility in bulls. *EDEC3* is overexpressed in testis and may play a role in spermiogenesis and oogenesis (https://www.genecards.org/). *LIN28B* is another gene that is overexpressed in testis (https://www.genecards.org/) and is reported to be associated with globozoospermia, which is a rare but severe cause of male infertility in mammals [56, 57]. Moreover, *CLDN20*, *AFGL1*, *TIAM2*, *RCN2*, *ODF3L1*, and *ARID3B* are other genes that overexpressed in testis (https://www.genecards.org/), suggesting a potential role for these genes in relation to the SM trait. However, FDR for SM trait was calculated 7.5%, so these candidate genes need to be validated by using a different dataset to confirm their role in bull fertility.

## Conclusions

We aimed to address: (i) which semen traits are more susceptible to inbreeding depression (ii) Whether the detected inbreeding depression is due to recent or ancient inbreeding (iii) Where the associated genes with these traits are in the genome. In summary, we found sperm concentration and sperm motility were unfavourably affected by inbreeding. The effect of recent inbreeding on semen traits was more harmful, probably because of genetic purging effect reducing its impact in older inbreeding. Our results provide novel insights into where genes causing inbreeding depression of semen traits are in the genome. Breeding companies may wish to consider avoiding homozygosity in these regions for potential artificial insemination sires. Validation of these regions and additional research into candidate genes located in these regions using larger sample sizes and denser markers may shed light on the molecular mechanisms and the causal variants underlying inbreeding depression of semen traits in dairy cattle.

## Supplementary Information

**Additional file 1:** **Table S1.** Genome-wide association with inbreeding depression of sperm concentration.

**Additional file 2:** **Table S2.** Genome-wide association with inbreeding depression of sperm motility.

**Additional file 3:** **Table S3.** Genes associated with inbreeding depression of sperm motility trait.

## Data Availability

The datasets analysed during the current study are available in the figshare repository, https://figshare.com/articles/semen_trait_gwas/7562510.

## Abbreviations

EV: Ejaculate volume
SC: Sperm concentration
SM: Sperm motility
SNP: Single nucleotide polymorphism
ROH: Runs of homozygosity
BTA: Bos Taurus autosomes
FDR: False discovery rate
F: Inbreeding
IBS: Identical by state
IBD: Identical by descent
AI: Artificial insemination
NRR: Non-return rate
MAF: Minor allele frequency
